# Librarians and information specialists as methodological peer-reviewers: a case-study of the International Journal of Health Governance

**DOI:** 10.1186/s41073-023-00142-4

**Published:** 2024-01-19

**Authors:** Irina Ibragimova, Helen Fulbright

**Affiliations:** 1 HealthConnect International, F. Fanceva 70, Zadar, 23000 Croatia; 2 Centre for Reviews and Dissemination, University of York, York, UK

**Keywords:** Health science librarians, Information specialists, Methodological peer-reviewers, Segmented peer-review, Evidence synthesis

## Abstract

**Background:**

Objectives of this study were to analyze the impact of including librarians and information specialist as methodological peer-reviewers. We sought to determine if and how librarians’ comments differed from subject peer-reviewers’; whether there were differences in the implementation of their recommendations; how this impacted editorial decision-making; and the perceived utility of librarian peer-review by librarians and authors.

**Methods:**

We used a mixed method approach, conducting a qualitative analysis of reviewer reports, author replies and editors’ decisions of submissions to the International Journal of Health Governance. Our content analysis categorized 16 thematic areas, so that methodological and subject peer-reviewers’ comments, decisions and rejection rates could be compared. Categories were based on the standard areas covered in peer-review (e.g., title, originality, etc.) as well as additional in-depth categories relating to the methodology (e.g., search strategy, reporting guidelines, etc.). We developed and used criteria to judge reviewers’ perspectives and code their comments.

We conducted two online multiple-choice surveys which were qualitatively analyzed: one of methodological peer-reviewers’ perceptions of peer-reviewing, the other of published authors’ views on the suggested revisions.

**Results:**

Methodological peer-reviewers assessed 13 literature reviews submitted between September 2020 and March 2023. 55 reviewer reports were collected: 25 from methodological peer-reviewers, 30 from subject peer-reviewers (mean: 4.2 reviews per manuscript). Methodological peer-reviewers made more comments on methodologies, with authors more likely to implement their changes (52 of 65 changes, vs. 51 of 82 by subject peer-reviewers); they were also more likely to reject submissions (seven vs. four times, respectively). Where there were differences in recommendations to editors, journal editors were more likely to follow methodological peer-reviewers (nine vs. three times, respectively). The survey of published authors (87.5% response rate) revealed four of seven found comments on methodologies helpful. Librarians’ survey responses (66.5% response rate) revealed those who conducted peer-reviews felt they improved quality of publications.

**Conclusions:**

Librarians can enhance evidence synthesis publications by ensuring methodologies have been conducted and reported appropriately. Their recommendations helped authors revise submissions and facilitated editorial decision-making. Further research could determine if sharing reviews with subject peer-reviewers and journal editors could benefit them in better understanding of evidence synthesis methodologies.

**Supplementary Information:**

The online version contains supplementary material available at 10.1186/s41073-023-00142-4.

## Aims and background

Many guidelines on conducting systematic, scoping and mapping reviews such as the JBI Manual for Evidence Synthesis; the Cochrane Handbook for Systematic Reviews of Interventions; and the Institute of Medicine Standards for Systematic Reviews, explicitly recommend inclusion of an experienced medical/healthcare librarian or information specialist as a team member [1–3]. This is because these types of evidence synthesis research require adherance to established methodology protocols, including systematic, transparent, and reproducible search strategies. There is sufficient evidence that, as expert searchers, librarians and information specialists (hereafter referred to as ‘librarians’) can increase the quality, methodology and reporting of searches conducted for systematic reviews when included as research team members [4–6]. There are even networks of librarians who work on systematic reviews, in Scandinavia as well as other countries [7]. Yet, these specialists are rarely invited by journal editors to peer-review evidence synthesis publications. A recent survey of 291 librarians revealed that 78% (228) have never been asked to peer-review systematic review manuscripts, even though 54% (*n* = 122) of those not yet asked would be prepared to perform this role [8].

The existence of specially developed tools such as the Peer Review of Electronic Search Strategies (PRESS) Evidence-Based Checklist, which assists scrutiny of search stratgies – ensuring these have been designed appropriately for the topic and avoid making common mistakes [9–11], highlights that librarians might already be applying some of these skills in self-evaluation of their own work, or in peer-reviewing the work of others. This is also supported by the use of platforms for librarians to share peer-reviewed search strategies. One such platform, developed by a group of expert searchers from various NHS libraries upon the appeal of Health Education England (HEE) [12], demonstrates that this is a widespread and respected practice.

Dinakaran and colleagues suggested “segmented peer-review” as a new process for reviewing multidisciplinary research submissions, as such papers present logistical and practical barriers to effective peer review [13]. In this process, while submitting their manuscripts, authors “explicitly identify each of the areas of expertise required to review the paper, direct the reviewer to the relevant portions of the manuscript, and suggest in-field reviewers” [13]. There is evidence that choosing peer-reviewers for specific tasks or with specific expertise (e.g., a statistical peer reviewer) can improve the quality of the final manuscript [14]. Nyhan and Grossetta Nardini have discussed the merits of this ‘segmented’ approach for peer-reviewing evidence synthesis submissions, since “few individual peer-reviewers have the requisite knowledge to evaluate all aspects of evidence synthesis manuscripts” [15]. As part of a segmented peer-review process, librarians could serve as methodological peer-reviewers (MPR), allowing their expertise to be utilized without having to be experts on the paper’s subject matter.

Although librarians have expressed interest in being methodological peer-reviewers, there has been limited investigation of the impact of librarians as peer-reviewers on published evidence synthesis research. We are aware of one ongoing trial evaluating the effect of using librarians and information specialists as methodological peer-reviewers on the quality of search reporting and risk of bias in searches for systematic reviews [16]. Additionally, Townsend and colleagues have compared the impact of librarian and non-librarian peer-reviewers on systematic reviews published in a set of medical journals in 2017, and investigated whether editors took guidance from the librarian peer-reviewers [17]. However, as this work is available as a dataset rather than a scholarly article, no written discussion or conclusion is available from these authors.

This paper aims to analyze the impact of librarians as methodological peer-reviewers on the peer-review process in one health sciences journal. The objectives of this study are to determine: if and how comments from methodological peer-reviewers differ from those of subject peer-reviewers (SPR); whether there are differences in the implementation of recommendations between MPRs and SPRs; how the inclusion of MPRs impacts editorial decision-making; and how librarians and authors perceived the utility of librarian peer review.

For the purpose of this study, ‘subject peer-reviewers’ refers to specialists registered in ScholarOne system as authors or reviewers with relevant areas of expertise.

## Methods

### Study design

Using a mixed method approach, we conducted a qualitative analysis of reviewer reports and author replies. We also conducted two surveys: of MPRs and published authors (which were also qualitatively analyzed). This approach allowed us to conduct a detailed study of evidence synthesis manuscripts submitted to the journal and to qualitatively analyze this data in addition to our survey responses. The use of multiple methods to collect and analyze data is encouraged and found to be mutually informative in case-study research to provide a synergistic and comprehensive view of the issue being studied [18]. This approach was also appropriate, as a central focus of the study is to answer how and why questions, without any influence exerted on those involved in the study [19]. Our study is reported in accordance with guidelines for organizational case-studies – a framework developed within the Health Services and Delivery Research program (part of the National Institute for Health Research) [20]. We also applied the Checklist for Reporting Results of Internet E-Surveys [21] and Standards for Reporting Qualitative Research [22]. All completed checklists are available in a Supplementary file.

### Call for librarians and information specialists in the International Journal of Health Governance

In June 2020, the Canadian Health Libraries Association, the European Association for Health Information and Libraries, the Health Libraries Australia section of the Australian Library and Information Association and the US Medical Library Association published a letter to the International Committee of Medical Journal Editors [23] to encourage journal editors to actively seek librarians as peer-reviewers for knowledge synthesis publications and to advocate for the recognition of their methodological expertise. This was especially important at this time, as the COVID-19 pandemic led to a substantial increase in submissions for publication. The letter also pointed journal editors to the Librarian Peer Reviewer Database [24], which connects librarians with expertise in evidence synthesis and journal editors in need of peer-reviewers with this knowledge. In response to this letter, the editor of the International Journal of Health Governance used a mailing list for members of the European Association for Health Information and Libraries to invite librarians with experience in systematic reviews to express interest in becoming methodological peer-reviewers.

The librarians who volunteered and performed methodological peer-analyses represented a broad range of medical institutions and organizations internationally: including royal colleges, university libraries, medical centers, and the National Health Service in the United Kingdom. As these organizations are likely to work on many different review types, a wealth of methodological knowledge and expertise was represented.

A guide for librarians as methodological peer-reviewers was compiled and distributed to them, following discussion of their specific needs in performing the role. The guide explained the step-by-step review process; stated that there was no obligation to assess all sections of manuscripts; and provided links to additional resources for librarians as peer-reviewers (*see* Supplementary file). The guide is revised annually and distributed to the journal’s methodological peer-reviewers.

The International Journal of Health Governance (IJHG) is a peer-reviewed journal concerned with the evolution of governance for health and healthcare systems and is indexed in the database Emerging Sources Citation Index on the Web of Science platform. The journal’s content includes empirical and theoretical papers that offer national perspectives, international comparisons, and global approaches. It publishes a variety of literature reviews (including narrative, systematic, scoping and mapping reviews), in addition to other publication types. The journal was previously published as Clinical Governance: An International Journal (2003–2015), and in addition to its name has undergone significant changes in its aim, scope, and editorial team since 2016. The journal applies a double-anonymized model of peer-review – this means the reviewer only interacts with the editor, and no information about the review process or editorial decision process is published. It is a hybrid type of access journal with two open access publishing paths (gold and green open access), that receives about 100–120 submissions annually, with 30 manuscripts published over 4 issues. For 2022 the impact factor for the journal was rated 1.3 by Clarivate Analytics.

### Content analysis of reviewer reports and author replies

This mixed methods study provides a content analysis of evidence synthesis manuscripts submitted to the International Journal of Health Governance from September 2020 to March 2023. Using the ScholarOne system, the journal editor extracted the reviewers’ reports and editors’ decisions alongside authors’ replies, as this data is not publicly available. To conduct a content analysis of peer-reviewers’ reports, we developed a system to categorize main thematic areas so that different groups of reviewers (methodological and subject peer-reviewers) could be compared against each other [25, 26]. To categorize the sections needed for our analysis, we applied the standard questions used by the journal in the peer-review process, covering the following sections of manuscripts: ‘title’, ‘structured abstract’, ‘originality’, ‘introduction/background’, ‘relation to existing literature’, ‘methodology’, ‘results’, ‘conclusions’, ‘implications for research’, ‘implications for practice/society’, ‘references’, and ‘quality of communication’. In addition to these categories, we also added more in-depth sections concerning methodologies: ‘manuscript type’, ‘review type’, ‘search strategy’, and ‘reporting guideline’ to collect more complete data on these important aspects, which can affect the quality of evidence synthesis publications when improperly conducted, applied, or presented [9, 27–29]. Overall, we used 16 categories for analysis (*see* Supplementary file for a detailed description of each category).

In addition to categorizing reviewers’ comments, we evaluated how a reviewer judged each individual section of a manuscript. Various approaches and tools, which have been developed to evaluate peer-review reports, suggest that annotation of reviewers’ comments as ‘positive’, ‘negative’ or ‘neutral’ could be used to analyze the reviewer’s perspective on different aspects of a paper [30]. We therefore developed our criteria to judge reviewers’ perspectives and coded comments as: ‘positive’, ‘negative’, ‘recommended change’, or ‘no comment’. As an example of the difference between ‘negative’ and ‘recommended change’, the description of a search strategy as flawed would be ‘negative’, in comparison to recommending additional database to be searched, which would be a ‘recommended change’.

Data for each manuscript (the reviewers’ comments, author replies, and editor decisions for all versions) was coded in a separate worksheet. All comments and replies were analyzed in relation to 16 categories, with each comment labelled as ‘positive’, ‘negative’, ‘recommended change’, or ‘no comment’ and each reply labelled as ‘accepted change’ or ‘declined change’. This allowed comparison of comments between MPRs and SPRs relating to the same sections of the same manuscripts. It also helped us assess which reviewer recommendations were accepted or declined by the authors, and to understand which changes were made in the manuscripts. All coded data was then combined in one worksheet (available in a Supplementary file). Coded data for each individual manuscript are available on the Zenodo platform (10.5281/zenodo.8418107).

### Comparing decisions recommended by MPRs and SPRs

To assess whether MPR recommendations impacted editorial decision-making, we looked at the gap between MPRs’ and SPRs’ suggested decisions to revise, accept or reject submitted manuscripts. We compared recommended decisions of the MPRs and SPRs for each manuscript version and then compared these with the editors’ decisions. Where there were differences in recommendations between MPRs and SPRs, we counted the number of times that editors made their decisions in agreement with either MPRs or SPRs. We also compared the reasons for rejecting manuscripts provided by MPRs and SPRs, and the number of confidential comments to the editor that they provided.

### Online surveys of authors and peer-reviewers

We collected qualitative evidence in the form of two online surveys, developed using SurveyPlanet, which were designed to take under 5 min. The surveys were set as ‘Completely Anonymous’ (thus not capturing any identifying information about participants, including IP address, locations, browser details and operating systems). Respondents were not able to change their answers or submit multiple responses, as cookies were used to assign a unique user-identifier to each client computer.

An online survey was offered by e-mail to all librarians who had registered their interest in being MPRs for the journal. The survey invitation included information on the study purpose, investigators, estimated length to complete the survey, anonymized data, and planned publication of results. No incentives were offered for participation. The survey was open for six weeks between February and March 2023. Librarians were asked two multiple-choice questions about their perceptions of their impact on the peer-reviewing process for the journal: one mandatory and one non-mandatory. There was also an open-ended question for comments. The survey also catered for responses from those who had not yet participated as MPRs and sought to understand any potential barriers to participation.

Another survey, using the same platform and approach, was offered to authors of evidence synthesis articles accepted for publication in the journal between August 2020 – May 2023. This was open for ten weeks between March and May 2023. This survey asked about the inclusion of librarians within their research teams, and their impressions of reviewers’ feedback on the methodology section of their submissions. It consisted of two mandatory multiple-choice questions and one open-ended question for comments. This allowed us to gauge whether librarians were included at any stage of their work, and whether authors found reviews from MPRs useful for revising the methodology sections of their manuscripts.

We did not ask the authors of rejected manuscripts for feedback, as MPRs provided negative comments for the methodology sections of these rejected manuscripts, rather than recommendations for change. This meant we would not be able to measure the impact of MPRs on their revisions. For this reason, the survey was sent only to the published authors.

The survey questions and additional information are available in a Supplementary file. An ethics exemption certificate for this study was obtained from the University of York Health Sciences Research Governance Committee.

## Results

Of 12 librarians who responded to the editor’s call and formed a group of MPRs for the IJHG, eight accepted invitations to review evidence synthesis manuscripts. Methodological peer-reviewers served as referees for 13 literature reviews submitted to the journal between September 2020 and March 2023: two qualitative evidence syntheses; four systematic, two scoping, one rapid evidence, and four narrative reviews. A total of 55 reviewer reports (25 from MPRs and 30 from SPRs) were collected from 13 manuscripts that underwent peer-review with a final editorial decision (mean: 4.2 reviews per manuscript). The distribution of these reports is presented in Table 1.

**Table 1:** Reviewer reports by manuscript version

Manuscript version	MPR reports (*n* = 25)	SPR reports (*n* = 30)
initial version (R0)	14	16
first revision (R1)	10	11
second revision (R2)	1	2
third revision (R3)	0	1

### Differences in comments from methodological and subject peer-reviewers

To investigate how MPR comments differed from SPR comments, we compared how MPRs and SPRs commented on the same sections of the same manuscript (Table 2). It should be noted that some of the categories used in the analysis were aspects such as ‘originality’, or ‘quality of communication’ (rather than specific sections of the manuscript). For simplicity, we will refer to all categories as ‘sections’.

 Table 2 includes data from reviewer reports where comments were available both from MPRs *and* SPRs for the same manuscript version. For MPRs, data was obtained from 23 reports and for 368 sections that could be commented on and, for SPRs, from 26 reports and for 416 sections. Our analysis of this data shows that sections with ‘positive’ feedback from MPRs (*n* = 97) received comments with a variety of perspectives from SPRs. Notably, SPRs’ comments on the same sections of these manuscripts were ‘positive’ in 52 of 104 cases (50%); ‘negative’ in 5 of 104 cases (5%); ‘recommended change’ in 15 of 104 cases (15%); and SPRs did not provide any comment for 32 of 104 Sect. (30%). For the 39 sections with ‘negative’ feedback from MPRs, similar judgment was received from SPRs in 6 of 51 cases (12%), and ‘positive’ feedback from SPRs in 24 of 51 cases (48%). We found that for 195 sections commented upon by MPRs (as positive, negative, or recommended change), SPRs provided comments on the same sections in only 74 of 223 cases overall (33%).

**Table 2:** MPRs and SPRs judgments when commenting on the same sections

MPR judged a section	*N* = 368	SPR judged the same section	*N* = 416
Positive	97	Positive	52 (50%)
		Negative	5 (5%)
		Recommended change	15 (15%)
		No comment	32 (30%)
		**Total**	104 (100%)
Negative	39	Negative	6 (12%)
		Positive	24 (48%)
		Recommended change	7 (13%)
		No comment	14 (27%)
		**Total**	51 (100%)
Recommended change	59	Recommended change	21 (31%)
		Positive	19 (28%)
		Negative	0
		No comment	28 (41%)
		**Total**	68 (100%)
No comment	173	No comment	143 (74%)
		Positive	24 (12.5%)
		Negative	1 (0.5%)
		Recommended change	25 (13%)
		**Total**	193 (100%)

We also analyzed whether MPRs vs. SPRs were more likely to comment on specific sections of manuscripts by comparing comments from all reports by MPRs (*n* = 25) and SPRs (*n* = 30) (*see* the table ‘Manuscript sections with comments from MPRs or SPRs’ in the Supplementary file). Our analysis revealed that three categories (‘review type’, ‘search strategy’, and ‘reporting guidelines’) were almost exclusively commented on by MPRs (in 76%, 84%, and 32% of 25 reports, respectively) and rarely by SPRs (in 26%, 26%, and 6% of 30 reports, respectively). MPRs were also more likely to comment on a manuscript’s conclusion compared with SPRs (80% vs 53%, respectively). In comparison, SPRs were more likely to comment on the originality of research compared with MPRs (96% and 76% respectively), and on implications for practice (66% and 48% respectively). For many other categories of comments, there were very similar numbers between MPRs and SPRs.

It should be noted that comments from SPRs and MPRs do not necessarily reflect the views of the journal editors. MPRs comments on ‘review type’ covered the following aspects:

- recommendation to include review type in the title;
- the rationale for choosing the specific review type;
- whether the review type as stated by author adheres to the requirements for conducting and reporting that type of evidence synthesis research;
- whether a search strategy was adequate for the stated review type;
- to explain (where relevant) which qualitative approach to synthesis was used (configurative, integrative, or aggregative);
- whether the methodology of evidence synthesis was appropriately described;
- recommendation to reference papers on the methodology for conducting that type of review (e.g., rapid evidence review, narrative review);
- described limitations of the evidence synthesis used; and.
- (where relevant), rated how the manuscript scored against the scale for assessment of that review type (e.g., the Scale for the Assessment of Narrative Review Articles).

In comparison, comments from SPRs on ‘review type’ covered fewer aspects:

- recommendation to provide a definition of a review type;
- described the bias inherent to some review types;
- stated that the manuscript is a scoping review and not a research study;
- recommendation to explain who performed the searches and assessed the quality of included studies; and.
- recommendation to reference papers on the methodology for conducting that type of review.

For comments made on search strategies, we found that MPRs provided more comments and were more detailed in terms of the recommendations provided (Table 3). In their reports, MPRs referred to “search strategy” 13 times and to “literature search” seven times, in comparison SPRs referred to “search strategy” or “search criteria” just five times.

**Table 3:** Search strategy components commented on by MPRs and SPRs

Search Strategy Component	MPR reports (*N* = 21)	SPR reports (*N* = 8)
Protocol registration	2	0
Inclusion/exclusion criteria	4	2
Sources (databases) searched	9	1
Platforms used (for databases)	1	0
Listing search strategy for each database	4	0
Rational for studies being excluded from review	2	2
MeSH terms included in search	6	1
Textwords included in search	4	2
Combination of MeSH and textwords	1	0
Basic or Advanced search (in a specific database)	3	0
Grey literature	3	1
Term truncation	6	0
Synonyms included in search strategy	2	0
Phrase searching	4	0
Reference manager used	1	0
Screening of search results	2	0
Date limit	2	2
Language/geographical limits	1	1
Sampling strategy (purposive, theoretical, comprehensive)	3	0
PICO and other frameworks for presenting research question	1	1
Limit by study type	1	0
Search syntax	1	0
Search not designed by an expert searcher	1	0
Search not documented by an expert searcher	1	0

In nine reports where MPRs commented on the use of reporting guidelines, MPRs either stated that: the Preferred Reporting Items for Systematic reviews and Meta-Analyses (PRISMA) guidelines were not used (*n* = 1); that they were referenced but used inappropriately (*n* = 5); or that they were used correctly (*n* = 1). MPRs also emphasized that in some cases PRISMA was incorrectly considered by authors to be a guideline for conducting systematic reviews (*n* = 2); that an out-of-date version of the guideline was used (*n* = 1); or recommended to adhere to PRISMA extension for scoping reviews (*n* = 1). In comparison, SPRs mentioned PRISMA twice, stating the need for providing the full title of the guidelines, and made recommendations to explain their compliance with PRISMA guidelines in more depth.

### Are there differences in the authors’ implementation of recommendations from SPRs versus MPRs?

To explore the impact of MPRs’ suggested revisions on publications, we analyzed the types of changes suggested by MPRs and SPRs, and which of these were implemented by authors. This information was based on reviewers’ reports and on authors’ replies (Table 4). Table 4 also includes the reasons MPRs and SPRs recommended rejection of the manuscript (where applicable).

**Table 4:** Changes/rejections recommended by MPRs and SPRs

Manuscript section	MPR suggested change	Author made change	SPR suggested change	Author made change	MPR suggested Reject or RR	SPR suggested Reject or RR
Title	3	2	2	1		
Manuscript type	0		1	0		
Abstract	3	2	4	3		
Originality	0		3	2		
Review type	1	0	1	0	1	
Search strategy	11	9	6	5	5	
Reporting guideline	5	4	1	0		
Background	1	1	6	3		
Relation to existing literature	5	4	10	6		
Methodology	8	7	8	5		1
Results	4	4	7	5		2
Conclusions	7	6	5	3		
Implication for research	4	3	7	5		
Implication for practice	4	3	6	6		
Communication	9	7	14	7	1	1
References	0		1	0		
**Total**	**65**	**52**	**82**	**51**	**7**	**4**

MPRs suggested changes to all sections of manuscripts, except ‘originality’, ‘manuscript type’ and ‘references’. The most frequently recommended changes related to three categories: ‘search strategy’, ‘methodology’, and ‘quality of communication’. Most of the suggested changes from MPRs were accepted and implemented by authors (52 of 65, 80%). In comparison, SPRs’ most frequent suggested changes were for: ‘quality of communication’, ‘relation to existing literature’ (if the authors presented an adequate summary of current research literature to provide context), and ‘methodology’ (not including search strategies and reporting guidelines). 51 of 82 changes suggested by SPRs (62%) were implemented by authors. Some recommended changes could not be implemented where manuscripts received an ‘accept’, ‘reject’, or ‘reject and resubmit’ decision from the editor. Reject and resubmit (RR) is recommended when research has potential but should be rewritten and resubmitted as a new manuscript (not a revision). For these above-mentioned reasons, one change (1.5%) recommended by an MPR and 14 changes (17%) recommended by SPRs could not be implemented.

We found that MPRs who recommended changes to the search strategies typically: explained more detailed techniques for searching databases; provided examples on how to describe and report reproducible search strategies; and explained which elements required by reporting guidelines were missing. In comparison, recommendations from SPRs on search strategies (in 6 of 30 reports), tended to be more general, mentioning the need for additional keywords and searches of grey literature to be included.

### How do MPRs’ comments impact editorial decision-making?

To assess whether MPR comments assisted editors in their decisions to accept or reject manuscripts for publication, we looked at the gap between MPRs’ and SPRs’ suggestions and compared these with the editors’ decisions (Table 5). It should be noted that in some cases, after an author submits a revision in response to a ‘minor revision’ decision, the manuscript might be accepted by the editor for publication without an additional round of reviews (so there are more editor decisions included in our analysis, than shown in Table 5). Table 5 includes decisions on different versions of each manuscript (R0 – initial version, R1 – first revision, R2 – second revision, R3 – third revision).

**Table 5:** MPRs’, SPRs’ and editors’ decisions

Manuscript Version	MPR decision (*n* = 25)	SPR decision (*n* = 30)	Editor decision	Editor agreed with MPR	Editor agreed with SPR
Manuscript 1 R0	Major	Minor	Major	yes	no
Manuscript 1 R1	Minor	Accept	Minor	yes	no
Manuscript 2 R0		Major/Major	Major		
Manuscript 2 R1	Minor	Major/Minor	Minor		
Manuscript 2 R2		Minor	Minor		
Manuscript 2 R3		Minor	Accept		
Manuscript 3 R0	Major	Accept	Major	yes	no
Manuscript 3 R1	Accept	RR/Minor	Minor	no	yes
Manuscript 3 R2	RR	RR	RR		
Manuscript 4 R0	Accept/Minor	Minor	Minor		
Manuscript 4 R1	Accept	Accept	Accept		
Manuscript 5 R0	Minor	Accept	Minor	yes	no
Manuscript 5 R1	Accept		Accept		
Manuscript 6 R0	Reject	Major	Reject	yes	no
Manuscript 7 R0	Reject	Accept/Minor	Minor	no	yes
Manuscript 7 R1	RR/RR	Accept/Minor	RR	yes	no
Manuscript 8 R0	Minor		Minor		
Manuscript 8 R1	Accept	Major	Minor	no	no
Manuscript 9 R0	Reject	Major/RR	Reject	yes	no
Manuscript 10 R0	Accept	Minor	Minor	no	yes
Manuscript 11 R0	Minor	Accept	Minor	yes	no
Manuscript 12 R0	Major	Major/Minor	Major		
Manuscript 12 R1	RR	RR	RR		
Manuscript 13 R0	Major/Major	Major	Major		
Manuscript 13 R1	Accept	Minor	Accept	yes	no

It was not always possible to find both an MPR and an SPR to review some of the original submissions (e.g., manuscripts 2 and 8), or some of the later, revised versions (e.g., manuscript 5). There were 20 ‘matched pairs’ of reviewer reports submitted by MPRs and SPRs for the same version of a specific manuscript (note that in some cases there were two MPRs and/or two SPRs reviewing the same version, as shown by the use of forward slash).

Between MPRs and SPRs there was low agreement on recommendations to ‘accept’ a manuscript: in six reports from MPRs and seven from SPRs there was agreement only in one case. However, there was higher agreement for the recommendation to ‘reject’ a manuscript (including ‘reject and resubmit’) - there were 7 MPR reports in agreement with 3 reports from SPRs, and 4 SPR reports in agreement with 3 reports from MPRs. In terms of the recommendation to revise a manuscript (with either major or minor revisions), there were 10 MPR reports in agreement with seven reports from SPRs, and 15 SPR reports in agreement with six reports from MPRs.

Out of 20 ‘matched pairs’ of reviewer reports submitted by MPRs and SPRs for the same version of a specifc manuscript, there were differences in recommendations in 13 cases. In these 13 cases, the editors made their decisions in agreement with MPRs in nine cases, and in agreement with SPRs in three cases. An ‘in-between’ decision was made in one case.

Although there were fewer reports from MPRs included in this case-study compared with SPRs, we found that MPRs were more likely to recommend the rejection of manuscripts (seven times vs four times, respectively) (*see* Table 4). Recommendations for rejection (either to ‘reject’ or to ‘reject and resubmit’) were based on the search strategies (*n* = 5), review type (*n* = 1), and the quality of communication (*n* = 1). In comparison, SPRs recommendations to reject manuscripts were based on the quality of communication (*n* = 1), the quality of results (*n* = 2), and on the methodology (*n* = 1). MPRs were also more likely to recommend rejection at an earlier stage of peer-review process (for manuscript initial version R0 – three cases, first revision R1 – three cases, second revision R2 – one case), in comparison with SPRs (R0 – one case, R1 – two cases, R2 – one case) (*see* Table 5). MPRs also provided more confidential comments to the editors (in 16 of 25 reports, 80%) compared with SPRs (in 10 of 30 reports, 33%).

Out of 13 literature reviews included in our analysis, five were rejected from publication in the journal. Both MPRs and SPRs recommended rejection for three of these five, whereas only MPRs recommended rejection for the remaining two.

### How do librarians and authors perceive the utility of librarian peer review?

In the results of our online survey of MPRs (8 responses from 12 contacted, 66.5% response rate), six of eight respondents felt that they made valuable contributions or had a positive impact as a methodological peer-reviewer for the journal by: facilitating the publication of higher quality research; raising awareness of the need for specialist input on search strategy development; providing criticism of submissions with detailed feedback and examples (where applicable) to improve the overall quality of work; providing feedback on how researchers should perform the searches that underpin their work; and noticing positive changes in the published articles that had been based on their specific recommendations. Two of eight respondents chose the answer ‘not applicable’, as they had not reviewed any evidence synthesis manuscripts for the journal at the time of the survey.

Our survey of the corresponding authors for the eight published manuscripts received seven responses (87.5% response rate). Respondents gave feedback on the suggested changes to their methodologies but were unaware if these were recommended by SPRs or MPRs. Four of seven respondents stated that the reviewers’ comments on their methodology sections were useful, while three of seven responded with ‘not applicable’ (though all received recommendations on changes to their methodology sections and subsequently revised them). Only one respondent provided enough detail in their feedback that could be related specifically to recommendations by an MPR.

## Discussion

Our analysis revealed significant differences both in the sections of manuscripts that were commented upon by MPRs vs. SPRs and in the reviewers’ perspectives. Notably, some important methodological aspects of manuscripts were commented on predominantly by MPRs and in greater detail – specifically, reporting guidelines and search strategies.

The input from MPRs on the use of reporting guidelines corresponds with findings from previous research: the inappropriate use of reporting guidelines for evidence synthesis publications is known to be a commonly occurring issue [27, 29, 31], despite the availability of detailed guidance [16], e.g., reporting guidelines for systematic reviews and related extensions such as PRISMA; PRISMA-ScR (extension for scoping reviews); and PRISMA-S (extension for reporting literature searches in systematic reviews) [32, 33]. An internal audit of all types of published articles in the IJHG (2020–2021) revealed that only four of 47 articles (8.5%) referenced and adhered to specific reporting guidelines [34].

Our results also support Rethlefsen’s hypothesis, that non-librarian peer-reviewers’ lack of expert knowledge in appraising search strategies and methodologies leads to poor reporting of searches and thereby increases the perceived risk of bias [16]. Within health care, there are many different review types used for evidence synthesis. 48 distinct review types are described in a paper published by Sutton and colleagues in 2019 [35], with new types emerging on a regular basis. This is further evidence of specialist subject knowledge that falls under the expertise of health science librarians.

Despite the clear benefits of librarians as methodological peer-reviews, there are not, at present, obvious categories of expertise for librarians to choose when registering as methodological peer-reviewers in ScholarOne Manuscripts submission system. This issue, also noted by Grossetta Nardini and colleagues in 2019 [8], could be resolved though journal providers’ updating their author and peer-reviewer registration processes.

Our study has also revealed significant differences between MPRs and SPRs when providing recommendations for editorial decisions. This corresponds with findings in other studies on agreement rate among pairs of reviewers in medical journals, which revealed higher agreement rate for recommendations to accept and revise, and much lower agreement rate for recommendation to reject [36, 37]. In comparison, our study showed a relatively higher rate of agreement to reject (43%) between the two groups of reviewers than was found by Kravitz and colleagues (7%) [36], and by Baethge and colleagues (31%) [37]. We also found a low rate of agreement to accept (in six reports from MPRs and seven from SPRs there was agreement only in one case).

Our study is the first to qualitatively analyze findings that editors were more likely to follow recommendations from MPRs than SPRs when assessing and making decisions on evidence synthesis manuscripts. This could be explained by MPRs usually recommending ‘stricter’ decisions than SPRs (in terms of: major versus minor revision; reject versus major revision; or minor revision versus accept), and by editors’ tendency to make stricter decisions as well. Where there were ‘stricter’ recommendations from SPRs, editors were also very likely to follow these recommendations. This interpretation is supported by research on editorial decisions in different medical journals, which showed that recommendation for rejection was the most influential for editorial decisions and was associated with a high rate of rejection, whereas recommendations for acceptance or minor revisions were also influential but to a lesser degree [36, 38, 39].

Another explanation could be that the MPRs had a better understanding of IJHG’s aims, scope, and expectations for peer-reviewers, compared with SPRs, due to maintaining more frequent contact with the journal editor during and after forming a group of MPRs. As shown by Glonti and colleagues [40], only a few editors of medical journals (mostly those who work for non-commercial publishers) regularly update the guidance provided to peer reviewers or send them customized messages to draw on their expertise. In comparison, editors working with commercial publishing groups stated that guidelines were standardized across the entire range of journals and therefore had broadly defined expectations from peer reviewers rather than specific guidance [40]. The IJHG’s dedicated guidance for MPRs (which was developed with their input), is regularly updated in order to communicate editors’ expectations effectively.

As MPRs were more likely to explain their recommendations in confidential comments to the editors, this could also have influenced the editors’ decisions.

In many biomedical journals, it is a common practice that the decision letter and all reviewer comments are sent to all authors and reviewers once the manuscript has received a decision. Research in nursing journals has shown that peer-reviewers find it helpful to view other peer reviewers’ comments [41]. Glonti and colleagues hypothesize that this practice could also serve as indirect training, offering reviewers an opportunity to learn from fellow reviewers [40, 42]. This practice is commonly performed at IJHG and, due to the specific characteristics of reviews by MPRs, has the potential to benefit SPRs and editors in their understanding of best practices in methodologies for evidence synthesis research, though further research is needed.

Librarians could also provide their input to journals’ submission guidelines (on aspects concerning methodologies). We found that our group of MPRs had specific recommendations for prospective authors, which were subsequently included in editorials published in the journal and could improve the quality of evidence synthesis manuscripts prior to submission. For instance, one recommendation was to advise prospective authors, when appropriate, to register their systematic review protocols with PROSPERO (an international database of prospectively registered systematic reviews in health and social care, welfare, public health, education, crime, justice, and international development, where there is a health-related outcome) [43], or with Open Science Framework [44]. Authors were advised to submit their protocols as Supplementary material with their evidence synthesis manuscript (if not published elsewhere previously), so that these can be made available to peer-reviewers, and later hosted on the publisher’s platform. In this respect, our study of librarians as MPRs has increased our understanding of their impact on evidence synthesis publications during the peer-review process and highlights their potential impact on future submissions as well.

Our survey of published authors did not increase our understanding of the impact of MPRs on manuscript revisions, as their answers did not provide sufficient detail. Moreover, three of seven respondents chose ‘not applicable’ as their answer to our question on the utility of feedback on their methodologies.

## Conclusions

As methodological peer-reviewers, librarians made valuable contributions to published evidence synthesis research in the IJHG. There were differences between MPRs’ and SPRs’ reports in terms of: reviewers’ perspectives; the sections commented upon; and their recommended changes. MPRs were more likely to comment on methodologies in comparison to SPRs, with authors more likely to implement these suggested revisions. This may suggest that comments from MPRs on methodological sections were clearer and more comprehensive, helping authors to revise their manuscripts. Furthermore, in addition to scrutinizing and suggesting improvements to methodologies, MPRs’ recommendations also helped authors to revise and improve their manuscripts across numerous other sections (‘title’, ‘abstract’, ‘search strategy’, ‘methodology’, ‘results’, ‘conclusions’, ‘implication for research/practice’, and ‘communication’). This is indicative of the broader contributions that librarians can make in the peer-review process.

Librarians’ recommendations also assisted the editors in their decisions to request revisions or accept or reject manuscripts for publication, due to both the volume of comments and the explanations given in confidential comments to the editors. Our findings present that librarians were more likely to reject manuscripts in comparison to SPRs with reasons for rejection relating to the methodologies in all but one instance. MPR recommendations to reject initial or early manuscript versions helped editors to make a ‘reject’ decision at an early stage without requesting further revisions, which saved time and reduced labor. Also in support of this claim is the evidence that editors tended to follow MPRs over SPRs, where there were differences of opinion.

Inclusion of librarians as MPRs could impact other aspects of conducting and presenting evidence synthesis research. For instance, recommendations to include the review type in the manuscript title will enhance their discoverability in databases searches. Moreover, raising authors’ awareness of the need for specialist input on search strategy development could lead to inclusion of librarians in research teams and impact the quality of future publications by the same authors. Methodological peer-reviewers’ positive perceptions of their impact on published research could encourage other librarians to seek such opportunities and support recognition of their methodological expertise.

The results of this mixed method study support the use of librarians and information specialists as MPRs and furthers our understanding of how published research can benefit from their inclusion. Our findings could therefore be used to improve existing journal policies and guidelines.

### Limitations

The limitations of our study are that we only assessed peer-reviewed reports for evidence synthesis publications over the course of 2.5 years in one health sciences journal. We therefore made conclusions based on a relatively small sample: 13 evidence synthesis manuscripts, 55 reviewer reports, 16 author replies, and 29 editors’ decisions. Only one author was directly involved in the coding and analysis of the data, and no formal analysis of the reliability of the coding was conducted.

Our survey questions were designed to be completed quickly and were only distributed to a small number of people (12 librarians and eight published authors). The responses we received from authors were often not detailed enough to draw conclusions.

### Supplementary Information

**Additional file 1.**

** Additional file 2.**

## Data Availability

All datasets are available at Zenodo (10.5281/zenodo.8418107).
