# Supplementary material for: Librarians and information specialists as methodological peer-reviewers: a case-study of the International Journal of Health Governance
Source: Res Integr Peer Rev. 2024 Jan 19;9:1. doi: 10.1186/s41073-023-00142-4 (PMC10797710; doi:10.1186/s41073-023-00142-4)
Supplement: Supplementary file 1 — Additional file 1. [file 41073_2023_142_MOESM1_ESM.docx]

**Additional file 1. Librarians and information specialists as methodological peer-reviewers: a case study of the International Journal of Health Governance.**

Contents

[**1.** **Guide for librarians as methodological peer-reviewers** 2](#_Toc146011651)

[**2.** **Categories of manuscript sections and aspects** 4](#_Toc146011652)

[**3.** **Manuscript sections with comments from MPRs or SPRs** 5](#_Toc146011653)

[**4.** **Survey of authors who published evidence synthesis articles in the IJHG. Survey questions** 6](#_Toc146011654)

[**5.** **Survey of methodological peer-reviewers. Survey questions** 7](#_Toc146011655)

[**6.** **Coded data for 13 evidence synthesis articles (reviewer reports and author replies)** 9](#_Toc146011656)

[**7.** **Standards for Reporting Qualitative Research (SRQR) Checklist** 10](#_Toc146011657)

[**8.** **Consensus standards for the reporting of organisational case studies. Checklist** 13](#_Toc146011658)

[**9.** **Checklist for Reporting Results of Internet E-Surveys (CHERRIES)** 15](#_Toc146011659)

[**10.** **HSRGC Ethics exemption certificate** 18](#_Toc146011660)

# **Guide for librarians as methodological peer-reviewers**

Dear colleagues,

as you have expressed an interest in peer-reviewing for the **International Journal of Health Governance (IJHG)**, please

1. **Register** on the journal web-site in ScholarOne system

<https://mc.manuscriptcentral.com/ijhg>

1. **When creating your profile** you need to choose at least five areas of expertise from the predefined list. You might consider the following categories:

- Research Methods
- Informatics
- Clinical governance < Health Service Quality Assurance
- Clinical guidelines < Health Service Quality Assurance
- Health Service Quality Assurance
- Evidence-based practice < Health Service Quality Assurance

1. **At some point you might recieve an invitation to referee** a manuscript that is labelled as "literature review" category. For now IGHG does not distinguish between different types of literature reviews, so it could be:

- a systematic review
- a mapping review
- a scoping review
- a narrative review
- a review with a bibliometric analysis
- an umbrella review

The invitation includes a structured abstract of the submitted manuscript. At that point you can accept or decline the invitation.

1. If you **accept the invitation**, you will recieve a message with a link to the full-text of the manuscript. When reviewing the manuscript you are asked to **assess the following aspects**:

- **Originalit**y: Does the paper contain new and significant information adequate to justify publication?
- **Relationship to Literature**: Does the paper demonstrate an adequate understanding of the relevant literature in the field and cite an appropriate range of literature sources?
- **Methodology**: Is the paper's argument built on an appropriate base of theory, concepts or other ideas? Has the research or equivalent intellectual work on which the paper is based been well designed? Are the methods employed appropriate?
- **Results**: Are results presented clearly and analysed appropriately? Do the conclusions adequately tie together the other elements of the paper?
- **Implications for research, practice and/or society**: Does the paper identify clearly any implications for research, practice and/or society? Does the paper bridge the gap between theory and practice? How can the research be used in practice (economic and commercial impact), in teaching, to influence public policy, in research (contributing to the body of knowledge)?
- **Quality of Communication**: Does the paper clearly express its case, measured against the technical language of the fields and the expected knowledge of the journal's readership? Has attention been paid to the clarity of expression and readability, such as sentence structure, jargon use, acronyms, etc.

You can assess not all, but only specific sections of manuscripts (related to Methodology). You can also provide additional comments to the editor and/or to the author.

1. Then you have to **make a recommendation** to the editor:

- **Accept**
- **Minor revision required** – that might be related to author guideline requirements, e.g. a slight reduction in word count; formatting changes, such as the labelling of tables or figures; further evidence of an understanding of the research literature in the field; or a slight elaboration on the research findings.
- **Major revision required** - to address flaws in the methodology; collect more data; conduct a more thorough analysis; or even adjust the research question to ensure the paper contributes something truly original to the body of work.
- **Reject and resubmit** – when the research has a potential but it should be rewritten and resubmitted as a new manuscript.
- **Reject**

Your comments and recommendations will be sent to the author anonymously. The editor can edit them before sending to the author. After editor makes a decision all referees receive a message with all comments and recommendations (also anonymised). If the editor recommend a revision you could be later invited to review the revised manuscript (but you can decline the invitation).

**Additional resources on peer-reviewing**

*Peer-reviewer hub*

[*https://www.emeraldgrouppublishing.com/how-to/how-guides-reviewers/peer-review-hub*](https://www.emeraldgrouppublishing.com/how-to/how-guides-reviewers/peer-review-hub)

*Being critical and constructive: a guide to peer reviewing for librarians*

<http://jmla.pitt.edu/ojs/jmla/article/view/100>

# **Categories of manuscript sections and aspects**

| Manuscript section/aspect | Guiding questions (for reviewers) |
| --- | --- |
| Title | Does it clearly describe the article and include the most important keywords? (Consider how you search for research articles.) Does it demonstrate the significance of the research and make sense? |
| Structured abstract | Have all mandatory fields been completed? Does the abstract accurately reflect the content of the article? |
| Originality | Does the paper contain new and significant information adequate to justify publication? |
| Introduction/Background | Does this describe what the author hoped to achieve and clearly articulate the research question? |
| Relation to existing literature | Does the paper demonstrate an adequate understanding of the relevant literature in the field and cite an appropriate range of literature sources? Is any significant work ignored? |
| Methodology | Is the paper's argument built on an appropriate base of theory, concepts, or other ideas? Has the research or equivalent intellectual work on which the paper is based been well designed? Are the methods employed appropriate? |
| Results | Are results presented clearly and analyzed appropriately? |
| Conclusions | Do the conclusions adequately tie together the other elements of the paper? Does the article support or contradict previous theories? |
| Implications for research | Does the paper identify clearly any implications for research (contributing to the body of knowledge)? |
| Implications for practice/society | Does the paper bridge the gap between theory and practice? How can the research be used in practice (economic and commercial impact), in teaching, to influence public policy? What is the impact upon society (influencing public attitudes, affecting quality of life)? Are these implications consistent with the findings and conclusions of the paper? |
| References | Are the referenced sources adequately cited in the paper? |
| Quality of Communication | Does the paper clearly express its case, measured against the technical language of the fields and the expected knowledge of the journal's readership? Has attention been paid to the clarity of expression and readability, such as sentence structure, jargon use, acronyms, etc.? |
| Manuscript type (category) | *Is the most adequate type (category) chosen for the paper?* |
| Review type | *Does the stated review type correspond to the established guidelines for conducting it?* |
| Search strategy | *Is the search strategy properly developed, executed, and reported?* |
| Reporting guideline | *Is an adequate reporting guideline referenced and properly used?* |

# **Manuscript sections with comments from MPRs or SPRs**

| **Manuscript Section** | **Reports with MPR comments (N=25)** | **Reports with SPR comments (N=30)** |
| --- | --- | --- |
| Title | 5 (20%) | 2 (6%) |
| Manuscript type | 0 | 1 (3%) |
| Abstract | 5 (20%) | 4 (13%) |
| Originality | 19 (76%) | 29 (96%) |
| Review type | 19 (76%) | 8 (26%) |
| Search strategy | 21 (84%) | 8 (26%) |
| Reporting guideline | 9 (32%) | 2 (6%) |
| Background | 2 (8%) | 7 (23%) |
| Relation to existing literature | 24 (96%) | 28 (93%) |
| Methodology | 24 (96%) | 26 (87%) |
| Results | 22 (88%) | 29 (96%) |
| Conclusions | 20 (80%) | 16 (53%) |
| Implications for research | 16 (64%) | 20 (72%) |
| Implications for practice | 12 (48%) | 20 (66%) |
| Communication | 22 (88%) | 27 (90%) |
| References | 2 (8%) | 1 (3%) |

# **Survey of authors who published evidence synthesis articles in the IJHG. Survey questions**

***Invitation letter.***

Dear colleagues,

We are working on a case-study about the role of librarians as methodological peer-reviewers for evidence synthesis publications.

We would appreciate if you participate in our survey as a corresponding author of one of the following articles published in the IJHG…The survey is anonymous and will take up to 5 minutes. It will be opened till May 30th. The link is…

***Survey questions***

The aim of this survey is to research how librarians as methodological peer reviewers are contributing to peer-review process and the quality of published evidence synthesis articles in the International Journal of Health Governance.

Q.1. Has a librarian/information specialist participated at any step of your work on the manuscript you published in the IJHG?*

a. No

b. Yes (please, specify)

Q.2. How helpful it was to receive feedback on the methodology section of your manuscript from our peer-reviewers?*

a. Not helpful

b. Not applicable

c. It was helpful (please, specify)

Q.3. Any other comment

# **Survey of methodological peer-reviewers. Survey questions**

***Invitation letter.***

Dear colleagues,

hope you are well.

We are working on a case-study on the impact of librarians as methodology peer-reviewers.

Would appreciate if you participate in our online survey (it is anonymous, and will take up to 5 minutes).

The link is… It will be opened till the end of March.

***Survey questions***.

The aim of this survey is to research how librarians and information specialists as methodological peer reviewers are contributing to peer-review process and the quality of published evidence synthesis articles in the International Journal of Health Governance.

Q.1. How do you think your involvement as a methodology peer reviewer has impacted the quality of research published in the IJHG?*

a. I think that I have made valuable contributions / a positive impact as a MPR for the IJHG

b. I don't think that I have made valuable contributions / had a positive impact as a MPR for the IJHG

c. I am uncertain of the value of my contributions as a MPR for the IJHG

d. Not applicable

e. Other (please, specify)

Q.2. If you selected options a-c for Question 1, why do you think this?

Q.3. If you previously expressed interest in being a librarian MPR but then didn’t participate, why was this?

a. My circumstances changed and I did not have time to do this

b. I didn't feel confident in my ability to do the work / didn't know how much work was involved

c. Not Applicable

d. Other (please; specify)

Q.4. Any other comments

# **Coded data for 13 evidence synthesis articles (reviewer reports and author replies)**

# **Standards for Reporting Qualitative Research (SRQR) Checklist**

|  |  |  |
| --- | --- | --- |
|  |  |  |
|  |  | **Page/line no(s).** |
| **Title and abstract** | |  |
|  | **Title** - Concise description of the nature and topic of the study Identifying the study as qualitative or indicating the approach (e.g., ethnography, grounded theory) or data collection methods (e.g., interview, focus group) is recommended | p.2 /line 22 |
|  | **Abstract** - Summary of key elements of the study using the abstract format of the intended publication; typically includes background, purpose, methods, results, and conclusions | p.2/line 25-57 |
|  |  |  |
| **Introduction** | |  |
|  | **Problem formulation** - Description and significance of the problem/phenomenon studied; review of relevant theory and empirical work; problem statement | p.3-5/line 61-104 |
|  | **Purpose or research questio**n - Purpose of the study and specific objectives or questions | p.5/line 105-110 |
|  |  |  |
| **Methods** | |  |
|  | **Qualitative approach and research paradigm** - Qualitative approach (e.g., ethnography, grounded theory, case study, phenomenology, narrative research) and guiding theory if appropriate; identifying the research paradigm (e.g., postpositivist, constructivist/ interpretivist) is also recommended; rationale** | p.6/line 115-128 |
|  | **Researcher characteristics and reflexivity** - Researchers’ characteristics that may influence the research, including personal attributes, qualifications/experience, relationship with participants, assumptions, and/or presuppositions; potential or actual interaction between researchers’ characteristics and the research questions, approach, methods, results, and/or transferability | p.8/line 171-173 |
|  | **Context** - Setting/site and salient contextual factors; rationale** | p.6-8/line 129-168 |
|  | **Sampling strategy** - How and why research participants, documents, or events were selected; criteria for deciding when no further sampling was necessary (e.g., sampling saturation); rationale** | p.8/line 170-171 |
|  | **Ethical issues pertaining to human subjects** - Documentation of approval by an appropriate ethics review board and participant consent, or explanation for lack thereof; other confidentiality and data security issues | p.12/line 243-245 |
|  | **Data collection methods** - Types of data collected; details of data collection procedures including (as appropriate) start and stop dates of data collection and analysis, iterative process, triangulation of sources/methods, and modification of procedures in response to evolving study findings; rationale** | p.10/line 213-245 |
|  | **Data collection instruments and technologies** - Description of instruments (e.g., interview guides, questionnaires) and devices (e.g., audio recorders) used for data collection; if/how the instrument(s) changed over the course of the study | Suppl. file |
|  | **Units of study** - Number and relevant characteristics of participants, documents, or events included in the study; level of participation (could be reported in results) | p.12/line 248-254 |
|  | **Data processing** - Methods for processing data prior to and during analysis, including transcription, data entry, data management and security, verification of data integrity, data coding, and anonymization/de-identification of excerpts | p.8-9/line 173-194 |
|  | **Data analysis** - Process by which inferences, themes, etc., were identified and developed, including the researchers involved in data analysis; usually references a specific paradigm or approach; rationale** | p.9-10 /line 195-212 |
|  | **Techniques to enhance trustworthiness** - Techniques to enhance trustworthiness and credibility of data analysis (e.g., member checking, audit trail, triangulation); rationale** |  |
|  |  |  |
| **Results/findings** | |  |
|  | **Synthesis and interpretation** - Main findings (e.g., interpretations, inferences, and themes); might include development of a theory or model, or integration with prior research or theory | p.12-22/line 258-414 |
|  | **Links to empirical data** - Evidence (e.g., quotes, field notes, text excerpts, photographs) to substantiate analytic findings |  |
|  |  |  |
| **Discussion** | |  |
|  | **Integration with prior work, implications, transferability, and contribution(s) to the field -** Short summary of main findings; explanation of how findings and conclusions connect to, support, elaborate on, or challenge conclusions of earlier scholarship; discussion of scope of application/generalizability; identification of unique contribution(s) to scholarship in a discipline or field | p.22-25 /line 415-483 |
|  | **Limitations** - Trustworthiness and limitations of findings | p.27/line 519-527 |
|  |  |  |
| **Other** | |  |
|  | **Conflicts of interest** - Potential sources of influence or perceived influence on study conduct and conclusions; how these were managed | p.28/line 533-535 |
|  | **Funding** - Sources of funding and other support; role of funders in data collection, interpretation, and reporting | p.28/line 536 |
|  |  |  |
|  |  |  |
|  |  |  |
|  |  |  |
|  | **The rationale should briefly discuss the justification for choosing that theory, approach, method, or technique rather than other options available, the assumptions and limitations implicit in those choices, and how those choices influence study conclusions and transferability. As appropriate, the rationale for several items might be discussed together. |  |
|  |  |  |
|  | **Reference:** |  |
|  | O'Brien BC, Harris IB, Beckman TJ, Reed DA, Cook DA. **Standards for reporting qualitative research: a synthesis of recommendations.** *Academic Medicine*, Vol. 89, No. 9 / Sept 2014  DOI: 10.1097/ACM.0000000000000388 |  |
|  |  |  |
|  |  |  |

# **Consensus standards for the reporting of organisational case studies. Checklist**

| **Reporting item** | **Page number on which item was reported** | **Page number of justification for not reporting** |
| --- | --- | --- |
| **Describing the design** | | |
| 1. Define the research as a case study | p.6 |  |
| 2. State the broad aims of the study | p.5 |  |
| 3. State the research question(s)/hypotheses | p.5 |  |
| 4. Identify the specific case(s) and justify the selection | p.8 |  |
| **Describing the data collection** | | |
| 5. Describe how data were collected | pp.8-11 |  |
| 6. Describe the sources of evidence used | pp.8-9 |  |
| 7. Describe any ethical considerations and obtainment of relevant approvals, access and permissions | p.12 |  |
| **Describing the data analysis** | | |
| 8. Describe the analysis methods | pp.8-10 |  |
| **Interpreting the results** | | |
| 9. Describe any inherent shortcomings in the design and analysis and how these might have influenced the findings | p.27 |  |
| 10. Consider the appropriateness of methods used for the question and subject matter and why it was that qualitative methods were appropriate | p.6 |  |
| 11. Discuss the data analysis | p.12-22 |  |
| 12. Ensure that the assertions are sound, neither over- nor under-interpreting the data | pp.22-25 |  |
| 13. State any caveats about the study | p.27 |  |

# **Checklist for Reporting Results of Internet E-Surveys (CHERRIES)**

| ***Checklist Item*** | ***Explanation*** | ***Page Number*** |
| --- | --- | --- |
| Describe survey design | Describe target population, sample frame. Is the sample a convenience sample? (In “open” surveys this is most likely.) | 10-11 |
| IRB approval | Mention whether the study has been approved by an IRB. | 12 |
| Informed consent | Describe the informed consent process. Where were the participants told the length of time of the survey, which data were stored and where and for how long, who the investigator was, and the purpose of the study? | Suppl. file |
| Data protection | If any personal information was collected or stored, describe what mechanisms were used to protect unauthorized access. |  |
| Development and testing | State how the survey was developed, including whether the usability and technical functionality of the electronic questionnaire had been tested before fielding the questionnaire. | 10 |
| Open survey versus closed survey | An “open survey” is a survey open for each visitor of a site, while a closed survey is only open to a sample which the investigator knows (password-protected survey). | 10 |
| Contact mode | Indicate whether or not the initial contact with the potential participants was made on the Internet. (Investigators may also send out questionnaires by mail and allow for Web-based data entry.) | 10 |
| Advertising the survey | How/where was the survey announced or advertised? Some examples are offline media (newspapers), or online (mailing lists – If yes, which ones?) or banner ads (Where were these banner ads posted and what did they look like?). It is important to know the wording of the announcement as it will heavily influence who chooses to participate. Ideally the survey announcement should be published as an appendix. | Suppl. file |
| Web/E-mail | State the type of e-survey (eg, one posted on a Web site, or one sent out through e-mail). If it is an e-mail survey, were the responses entered manually into a database, or was there an automatic method for capturing responses? | 10 |
| Context | Describe the Web site (for mailing list/newsgroup) in which the survey was posted. What is the Web site about, who is visiting it, what are visitors normally looking for? Discuss to what degree the content of the Web site could pre-select the sample or influence the results. For example, a survey about vaccination on a anti-immunization Web site will have different results from a Web survey conducted on a government Web site |  |
| Mandatory/voluntary | Was it a mandatory survey to be filled in by every visitor who wanted to enter the Web site, or was it a voluntary survey? |  |
| Incentives | Were any incentives offered (eg, monetary, prizes, or non-monetary incentives such as an offer to provide the survey results)? |  |
| Time/Date | In what timeframe were the data collected? | 11 |
| Randomization of items or questionnaires | To prevent biases items can be randomized or alternated. |  |
| Adaptive questioning | Use adaptive questioning (certain items, or only conditionally displayed based on responses to other items) to reduce number and complexity of the questions. |  |
| Number of Items | What was the number of questionnaire items per page? The number of items is an important factor for the completion rate. | Suppl. file |
| Number of screens (pages) | Over how many pages was the questionnaire distributed? The number of items is an important factor for the completion rate. |  |
| Completeness check | It is technically possible to do consistency or completeness checks before the questionnaire is submitted. Was this done, and if “yes”, how (usually JAVAScript)? An alternative is to check for completeness after the questionnaire has been submitted (and highlight mandatory items). If this has been done, it should be reported. All items should provide a non-response option such as “not applicable” or “rather not say”, and selection of one response option should be enforced. | Suppl.file |
| Review step | State whether respondents were able to review and change their answers (eg, through a Back button or a Review step which displays a summary of the responses and asks the respondents if they are correct). | 10 |
| Unique site visitor | If you provide view rates or participation rates, you need to define how you determined a unique visitor. There are different techniques available, based on IP addresses or cookies or both. |  |
| View rate (Ratio of unique survey visitors/unique site visitors) | Requires counting unique visitors to the first page of the survey, divided by the number of unique site visitors (not page views!). It is not unusual to have view rates of less than 0.1 % if the survey is voluntary. |  |
| Participation rate (Ratio of unique visitors who agreed to participate/unique first survey page visitors) | Count the unique number of people who filled in the first survey page (or agreed to participate, for example by checking a checkbox), divided by visitors who visit the first page of the survey (or the informed consents page, if present). This can also be called “recruitment” rate. |  |
| Completion rate (Ratio of users who finished the survey/users who agreed to participate) | The number of people submitting the last questionnaire page, divided by the number of people who agreed to participate (or submitted the first survey page). This is only relevant if there is a separate “informed consent” page or if the survey goes over several pages. This is a measure for attrition. Note that “completion” can involve leaving questionnaire items blank. This is not a measure for how completely questionnaires were filled in. (If you need a measure for this, use the word “completeness rate”.) | p.21-22 |
| Cookies used | Indicate whether cookies were used to assign a unique user identifier to each client computer. If so, mention the page on which the cookie was set and read, and how long the cookie was valid. Were duplicate entries avoided by preventing users access to the survey twice; or were duplicate database entries having the same user ID eliminated before analysis? In the latter case, which entries were kept for analysis (eg, the first entry or the most recent)? | 10 |
| IP check | Indicate whether the IP address of the client computer was used to identify potential duplicate entries from the same user. If so, mention the period of time for which no two entries from the same IP address were allowed (eg, 24 hours). Were duplicate entries avoided by preventing users with the same IP address access to the survey twice; or were duplicate database entries having the same IP address within a given period of time eliminated before analysis? If the latter, which entries were kept for analysis (eg, the first entry or the most recent)? |  |
| Log file analysis | Indicate whether other techniques to analyze the log file for identification of multiple entries were used. If so, please describe. |  |
| Registration | In “closed” (non-open) surveys, users need to login first and it is easier to prevent duplicate entries from the same user. Describe how this was done. For example, was the survey never displayed a second time once the user had filled it in, or was the username stored together with the survey results and later eliminated? If the latter, which entries were kept for analysis (eg, the first entry or the most recent)? |  |
| Handling of incomplete questionnaires | Were only completed questionnaires analyzed? Were questionnaires which terminated early (where, for example, users did not go through all questionnaire pages) also analyzed? |  |
| Questionnaires submitted with an atypical timestamp | Some investigators may measure the time people needed to fill in a questionnaire and exclude questionnaires that were submitted too soon. Specify the timeframe that was used as a cut-off point, and describe how this point was determined. |  |
| Statistical correction | Indicate whether any methods such as weighting of items or propensity scores have been used to adjust for the non-representative sample; if so, please describe the methods. |  |

# **HSRGC Ethics exemption certificate**

| 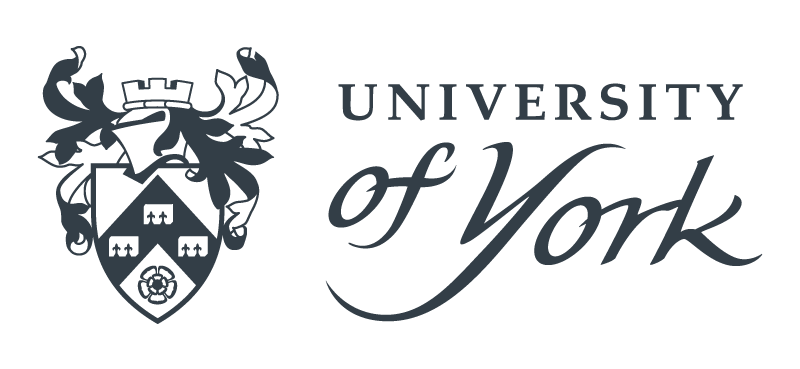  25 July 2023 | Department of  Health Sciences  c/o Department of Philosophy  Heslington  York YO10 5DD  Telephone (01904) 323253/1  E-mail [stephen.holland@york.ac.uk](mailto:stephen.holland@york.ac.uk)  Prof Stephen Holland  **Chair, Health Sciences Research Governance Committee**  www.york.ac.uk/healthsciences |
| --- | --- |

Dr. Helen Fulbright
Information Specialist / Research Fellow
Centre for Reviews and Dissemination
University of York
York
YO10 5DD

Dear Helen

***Re: Librarians as methodology peer reviewers***

Thank you for informing me of your study and for providing further details. In light of this information, I am writing to confirm that the study does not require review/approval by the Health Sciences’ Research Governance Committee. This is because the study is ethically very low risk and raises no substantive governance issues; the information is fully anonymous, and the aim of the study is to improve the peer review process as opposed to generating generalizable knowledge.

Yours sincerely


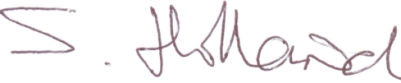


**Stephen Holland**

Chair: HSRGC
