# Supplementary material for: Librarians and information specialists as methodological peer-reviewers: a case-study of the International Journal of Health Governance
Source: Res Integr Peer Rev. 2024 Jan 19;9:1. doi: 10.1186/s41073-023-00142-4 (PMC10797710; doi:10.1186/s41073-023-00142-4)
Supplement: Supplementary file 2 — Additional file 2. [file 41073_2023_142_MOESM2_ESM.docx]

**Librarians and information specialists as methodological peer-reviewers: a case-study of the International Journal of Health Governance. Coded data for each manuscript**

**MPR** – methodological peer-reviewer (MPR1 – first methodological peer-reviewer, if there were more than one for the same version)

**SPR** – subject peer-reviewer (SPR1 – first subject peer-reviewer, if there were more than one for the same version)

**R0** – initial version of submitted manuscript

**R1** – first revision

**R2** – second revision

**R3** – third revision

reviewer recommended change - **C**

reviewer provided negative comment ­--

reviewer provided positive comment **+**

author accepted change - **C**

**Major –** major revision

**Minor –** minor revision

**RR**- reject and resubmit

| **Manuscript 1.** | **MPR**  **R0** | **SPR**  **R0** | **Editor**  **R0** | **Author**  **R1** | **MPR**  **R1** | **SPR**  **R1** | **Editor**  **R1** | **Author**  **R2** | **Editor**  **R2** |
| --- | --- | --- | --- | --- | --- | --- | --- | --- | --- |
| **Title** | **c** |  |  | **c** | **+** |  |  |  |  |
| **Article type** |  |  |  |  |  |  |  |  |  |
| **Abstract** |  |  |  |  |  |  |  |  |  |
| **Originality** | **+** | **+** |  |  | **+** | **+** |  |  |  |
| **Type of review** | **+** |  |  |  | **+** |  |  |  |  |
| **Search strategy** | **c** |  |  | **c** | **c** |  |  |  |  |
| **Reporting Guideline** |  |  |  | **c** |  |  |  |  |  |
| **Background** |  |  |  |  |  |  |  |  |  |
| **Related to literature** | **+** | **+** |  |  | **+** | **+** |  |  |  |
| **Methodology** | **c** | **+** |  | **c** | **c** | **+** |  |  |  |
| **Results** | **+** | **+** |  |  | **+** | **+** |  |  |  |
| **Conclusions** | **c** |  |  | **c** | **+** |  |  |  |  |
| **Implication for research** | **c** | **c** |  | **c** | **+** | **+** |  |  |  |
| **Implication for practice/society** | **+** |  |  |  |  |  |  |  |  |
| **Communication** | **c** | **+** |  | **c** | **c** | **+** |  | **c** |  |
| **References** | **+** |  |  |  |  |  |  |  |  |
| **Comments to editor** | **yes** | **no** |  |  | **yes** | **no** |  |  |  |
| **Comments to author** | **yes** | **no** |  |  | **yes** | **no** |  |  |  |
| **Decision** | **Major** | **Minor** | **Major** |  | **Minor** | **Accept** | **Minor** |  | **Accept** |

| **Manuscript 2.** | **SPR**  **R0** | **SPR**  **R0** | **Editor**  **R0** | **Author**  **R1** | **SPR**  **R1** | **SPR**  **R1** | **MPR**  **R1** | **Editor**  **R1** | **Author**  **R2** | **SPR**  **R2** | **Editor**  **R2** | **Author**  **R3** | **SPR**  **R3** | **Editor**  **R3** |
| --- | --- | --- | --- | --- | --- | --- | --- | --- | --- | --- | --- | --- | --- | --- |
| **Title** |  | **c** |  | **c** |  |  |  |  |  |  |  |  |  |  |
| **Article type** |  |  |  |  |  |  |  |  |  |  |  |  |  |  |
| **Abstract** | **c** |  |  | **c** |  | **c** |  |  | **c** |  |  |  |  |  |
| **Originality** | **+** | **+** |  |  | **+** | **+** | **+** |  |  | **+** |  |  | **+** |  |
| **Type of review** | **+** | **-** |  |  |  |  |  |  |  |  |  |  |  |  |
| **Search strategy** |  | **-** |  | **c** | **c** |  |  |  | **c** |  |  |  |  |  |
| **Reporting Guideline** |  |  |  |  |  |  |  |  |  |  |  |  |  |  |
| **Background** | **c** |  |  | **c** |  |  |  |  |  |  |  |  |  |  |
| **Related to literature** | **+** | **c** |  | **c** | **+** | **+** | **+** |  |  | **c** |  | **c** | **+** |  |
| **Methodology** | **c** | **c** |  | **c** | **+** | **+** | **+** |  |  | **c** |  | **c** | **+** |  |
| **Results** | **c** | **c** |  | **c** | **c** | **+** | **+** |  | **c** | **+** |  |  |  |  |
| **Conclusions** | **c** |  |  | **c** |  | **+** |  |  |  | **c** |  |  |  |  |
| **Implication for research** | **-** |  |  |  | **c** | **+** | **+** |  | **c** |  |  |  |  |  |
| **Implication for practice/society** | **-** |  |  |  | **c** | **+** |  |  | **c** |  |  |  |  |  |
| **Communication** | **c** | **c** |  | **c** | **c** | **+** | **c** |  | **c** | **c** |  | **c** | **c** |  |
| **References** |  |  |  |  |  |  |  |  |  |  |  |  |  |  |
| **Comments to editor** | **no** | **no** |  |  | **yes** | **no** | **no** |  |  | **yes** |  |  | **yes** |  |
| **Comments to author** | **yes** | **yes** |  |  | **yes** | **no** | **no** |  |  | **yes** |  |  | **no** |  |
| **Decision** | **Major** | **Major** | **Major** |  | **Major** | **Minor** | **Minor** | **Minor** |  | **Minor** | **Minor** |  | **Minor** | **Accept** |

| **Manuscript 3.** | **MPR**  **R0** | **SPR**  **R0** | **Editor**  **R0** | **Author**  **R1** | **MPR**  **R1** | **SPR**  **R1** | **SPR**  **R1** | **Editor**  **R1** | **Author**  **R2** | **MPR**  **R2** | **SPR**  **R2** | **Editor**  **R2** |
| --- | --- | --- | --- | --- | --- | --- | --- | --- | --- | --- | --- | --- |
| **Title** |  |  |  |  |  | **c** |  |  |  |  |  |  |
| **Article type** |  |  |  |  |  | **c** |  |  |  |  |  |  |
| **Abstract** |  |  |  |  |  |  |  |  |  |  |  |  |
| **Originality** | **+** | **+** |  |  | **+** | **c** | **+** |  | **c** | **+** | **+** |  |
| **Type of review** |  |  |  |  | **+** |  | **+** |  |  |  |  |  |
| **Search strategy** | **+** |  |  |  | **c** |  | **c** |  | **c** | **+** |  |  |
| **Reporting Guideline** |  |  |  |  |  |  |  |  |  |  |  |  |
| **Background** |  |  |  |  | **+** | **-** |  |  |  |  |  |  |
| **Related to literature** | **+** | **+** |  | **c** | **+** |  | **c** |  | **c** | **c** | **c** |  |
| **Methodology** | **c** | **+** |  | **c** | **+** | **-** | **c** |  |  |  | **+** |  |
| **Results** | **c** | **+** |  | **c** | **+** | **-** | **c** |  |  |  | **+** |  |
| **Conclusions** | **c** | **+** |  | **c** | **+** | **-** |  |  |  |  | **+** |  |
| **Implication for research** |  | **+** |  |  |  |  |  |  |  |  |  |  |
| **Implication for practice/society** | **c** |  |  | **c** |  |  |  |  |  |  |  |  |
| **Communication** | **-** | **+** |  | **c** | **+** |  | **c** |  | **c** | **-** | **-** |  |
| **References** |  |  |  |  |  |  |  |  |  |  |  |  |
| **Comments to editor** | **yes** | **no** |  |  | **no** | **yes** | **no** |  |  | **yes** | **no** |  |
| **Comments to author** | **yes** | **no** |  |  | **no** | **yes** | **yes** |  |  | **no** | **no** |  |
| **Decision** | **Major** | **Accept** | **Major** |  | **Accept** | **RR** | **Minor** | **Minor** |  | **RR** | **RR** | **RR** |

| **Manuscript 4.** | **MPR**  **R0** | **MPR**  **R0** | **SPR**  **R0** | **Editor**  **R0** | **Author**  **R1** | **MPR**  **R1** | **SPR**  **R1** | **Editor**  **R1** |
| --- | --- | --- | --- | --- | --- | --- | --- | --- |
| **Title** |  |  |  |  |  |  |  |  |
| **Article type** |  |  |  |  |  |  |  |  |
| **Abstract** |  |  |  |  |  |  |  |  |
| **Originality** | **+** |  | **+** |  |  | **+** | **+** |  |
| **Type of review** | **+** | **+** |  |  |  |  |  |  |
| **Search strategy** | **c** | **c** | **c** |  | **SPR - no** change  **MPR сс** | **+** |  |  |
| **Reporting Guideline** |  | **+** |  |  |  |  |  |  |
| **Background** |  |  |  |  |  |  |  |  |
| **Related to literature** | **+** | **c** | **c** |  | **c** | **+** | **+** |  |
| **Methodology** | **+** | **+** | **с** |  | **c** | **+** | **+** |  |
| **Results** | **+** | **+** | **+** |  |  | **+** | **+** |  |
| **Conclusions** | **c** | **-** |  |  | **c** | **+** | **+** |  |
| **Implication for research** | **+** | **-** | **c** |  |  |  | **+** |  |
| **Implication for practice/society** |  |  | **c** |  | **c** |  | **+** |  |
| **Communication** | **+** | **+** | **+** |  |  | **+** |  |  |
| **References** |  |  |  |  |  |  |  |  |
| **Comments to editor** | **yes** | **no** | **no** |  |  | **no** | **no** |  |
| **Comments to author** | **no** | **no** | **no** |  |  | **yes** | **no** |  |
| **Decision** | **Accept** | **Minor** | **Minor** | **Minor** |  | **Accept** | **Accept** | **Accept** |

| **Manuscript 5.** | **MPR**  **R0** | **SPR**  **R0** | **Editor**  **R0** | **Author**  **R1** | **MPR**  **R1** | **Editor**  **R1** |
| --- | --- | --- | --- | --- | --- | --- |
| **Title** | **c** |  |  | **c** | **+** |  |
| **Article type** |  |  |  |  |  |  |
| **Abstract** | **c** |  |  | **c** | **+** |  |
| **Originality** | **+** | **+** |  |  | **+** |  |
| **Type of review** | **+** | **+** |  |  | **+** |  |
| **Search strategy** | **c** |  |  | **c** | **+** |  |
| **Reporting Guideline** |  |  |  |  |  |  |
| **Background** |  |  |  |  |  |  |
| **Related to literature** | **+** | **+** |  |  | **+** |  |
| **Methodology** | **c** | **+** |  | **c** | **+** |  |
| **Results** | **c** | **+** |  | **с** | **+** |  |
| **Conclusions** | **c** | **+** |  | **c** | **+** |  |
| **Implication for research** | **c** | **+** |  | **c** | **+** |  |
| **Implication for practice/society** | **c** | **+** |  | **c** | **+** |  |
| **Communication** | **c** | **+** |  | **c** | **+** |  |
| **References** |  |  |  |  |  |  |
| **Comments to editor** | **yes** | **no** |  |  | **yes** |  |
| **Comments to author** | **yes** | **no** |  |  | **yes** |  |
| **Decision** | **Minor** | **Accept** | **Minor** |  | **Accept** | **Accept** |

| **Manuscript 6.** | **MPR**  **R0** | **SPR**  **R0** | **Editor**  **R0** |
| --- | --- | --- | --- |
| **Title** |  |  |  |
| **Article type** |  |  |  |
| **Abstract** |  | **c** |  |
| **Originality** | **-** | **+** |  |
| **Type of review** | **-** | **+** |  |
| **Search strategy** | **-** | **-** |  |
| **Reporting Guideline** | **-** |  |  |
| **Background** |  | **c** |  |
| **Related to literature** | **-** | **+** |  |
| **Methodology** | **-** | **-** |  |
| **Results** | **-** | **+** |  |
| **Conclusions** |  | **+** |  |
| **Implication for research** | **-** | **+** |  |
| **Implication for practice/society** |  |  |  |
| **Communication** | **+** | **+** |  |
| **References** | **-** |  |  |
| **Comments to editor** | **yes** | **yes** |  |
| **Comments to author** | **no** | **yes** |  |
| **Decision** | **Reject** | **Major** | **Reject** |

| **Manuscript 7.** | **MPR**  **R0** | **SPR1**  **R0** | **SPR2**  **R0** | **Editor**  **R0** | **Author**  **R1** | **MPR1**  **R1** | **MPR2**  **R1** | **SPR1**  **R1** | **SPR2**  **R1** | **Editor**  **R1** |
| --- | --- | --- | --- | --- | --- | --- | --- | --- | --- | --- |
| **Title** |  |  |  |  |  |  |  |  |  |  |
| **Article type** |  |  |  |  |  |  |  |  |  |  |
| **Abstract** |  |  |  |  |  |  |  |  |  |  |
| **Originality** | **-** | **+** | **+** |  |  |  |  | **+** | **+** |  |
| **Type of review** | **-** |  |  |  |  | **-** | **-** |  |  |  |
| **Search strategy** |  |  |  |  | **c** | **-** | **-** |  |  |  |
| **Reporting Guideline** |  |  |  |  | **c** | **-** | **-** |  |  |  |
| **Background** |  | **c** |  |  |  |  |  |  |  |  |
| **Related to literature** | **-** | **+** | **+** |  | **c** | **-** | **-** | **+** | **+** |  |
| **Methodology** | **-** | **+** | **+** |  | **c** | **-** | **-** | **+** | **+** |  |
| **Results** | **-** | **+** | **+** |  |  |  |  | **+** | **+** |  |
| **Conclusions** |  |  |  |  |  |  |  | **+** | **+** |  |
| **Implication for research** | **-** | **+** | **+** |  |  |  |  | **+** | **+** |  |
| **Implication for practice/society** | **-** | **+** | **+** |  |  |  |  | **+** | **+** |  |
| **Communication** | **-** | **+** | **c** |  |  |  |  | **+** | **c** |  |
| **References** |  |  | **c** |  |  |  |  |  |  |  |
| **Comments to editor** | **yes** | **yes** | **no** |  |  | **no** | **no** | **yes** | **no** |  |
| **Comments to author** | **no** | **yes** | **yes** |  |  | **no** | **no** | **yes** | **yes** |  |
| **Decision** | **Reject** | **Accept** | **Minor** | **Minor** |  | **RR** | **RR** | **Accept** | **Minor** | **RR** |

| **Manuscript 8.** | **MPR**  **R0** | **Editor**  **R0** | **Author**  **R1** | **MPR**  **R1** | **SPR**  **R1** | **Editor**  **R1** | **Author**  **R2** | **Editor**  **R2** |
| --- | --- | --- | --- | --- | --- | --- | --- | --- |
| **Title** |  |  |  |  |  |  |  |  |
| **Article type** |  |  |  |  |  |  |  |  |
| **Abstract** |  |  |  |  |  |  |  |  |
| **Originality** | **+** |  |  | **+** | **+** |  |  |  |
| **Type of review** | **+** |  |  | **+** |  |  |  |  |
| **Search strategy** | **c** |  | **c** | **+** |  |  |  |  |
| **Reporting Guideline** |  |  |  |  |  |  |  |  |
| **Background** |  |  |  |  | **c** |  | **c** |  |
| **Related to literature** | **+** |  |  | **+** | **+** |  |  |  |
| **Methodology** | **c** |  | **c** | **+** | **+** |  |  |  |
| **Results** | **+** |  |  | **+** | **c** |  | **c** |  |
| **Conclusions** | **c** |  | **c** | **+** | **c** |  | **c** |  |
| **Implication for research** | **c** |  |  |  | **c** |  | **c** |  |
| **Implication for practice/society** | **c** |  |  |  | **c** |  | **c** |  |
| **Communication** | **c** |  | **c** | **c** | **c** |  | **c** |  |
| **References** | **+** |  |  |  |  |  |  |  |
| **Comments to editor** | **yes** |  |  | **yes** | **yes** |  |  |  |
| **Comments to author** | **yes** |  |  | **yes** | **yes** |  |  |  |
| **Decision** | **Minor** | **Minor** |  | **Accept** | **Major** | **Minor** |  | **Accept** |

| **Manuscript 9.** | **MPR**  **R0** | **SPR1**  **R0** | **SPR2**  **R0** | **Editor**  **R0** |
| --- | --- | --- | --- | --- |
| **Title** |  |  |  |  |
| **Article type** |  |  |  |  |
| **Abstract** |  |  |  |  |
| **Originality** | **-** | **c** | **+** |  |
| **Type of review** |  |  |  |  |
| **Search strategy** | **-** |  |  |  |
| **Reporting Guideline** |  |  |  |  |
| **Background** |  |  |  |  |
| **Related to literature** | **-** | **c** | **c** |  |
| **Methodology** | **-** | **c** |  |  |
| **Results** | **-** | **-** | **-** |  |
| **Conclusions** |  | **c** | **c** |  |
| **Implication for research** | **-** | **+** | **c** |  |
| **Implication for practice/society** |  | **+** | **c** |  |
| **Communication** | **c** | **c** | **c** |  |
| **References** |  |  |  |  |
| **Comments to editor** | **yes** | **no** | **no** |  |
| **Comments to author** | **no** | **yes** | **no** |  |
| **Decision** | **Reject** | **Major** | **RR** | **Reject** |

| **Manuscript 10.** | **MPR**  **R0** | **SPR**  **R0** | **Editor**  **R0** | **Author**  **R1** | **Editor**  **R1** |
| --- | --- | --- | --- | --- | --- |
| **Title** |  |  |  |  |  |
| **Article type** |  |  |  |  |  |
| **Abstract** |  |  |  |  |  |
| **Originality** | **+** | **+** |  |  |  |
| **Type of review** | **+** |  |  |  |  |
| **Search strategy** | **+** |  |  |  |  |
| **Reporting Guideline** |  |  |  |  |  |
| **Background** |  |  |  |  |  |
| **Related to literature** | **+** | **+** |  |  |  |
| **Methodology** | **c** |  |  | **c** |  |
| **Results** | **+** | **+** |  |  |  |
| **Conclusions** | **+** |  |  |  |  |
| **Implication for research** | **+** | **+** |  |  |  |
| **Implication for practice/society** | **+** | **c** |  | **c** |  |
| **Communication** | **+** | **+** |  |  |  |
| **References** |  |  |  |  |  |
| **Comments to editor** | **no** | **no** |  |  |  |
| **Comments to author** | **no** | **yes** |  |  |  |
| **Decision** | **Accept** | **Minor** | **Minor** |  | **Accept** |

| **Manuscript 11.** | **MPR**  **R0** | **SPR**  **R0** | **Editor**  **R0** | **Author**  **R1** | **Editor**  **R1** |
| --- | --- | --- | --- | --- | --- |
| **Title** |  |  |  |  |  |
| **Article type** |  |  |  |  |  |
| **Abstract** | **c** |  |  | **c** |  |
| **Originality** | **+** | **+** |  |  |  |
| **Type of review** | **+** |  |  |  |  |
| **Search strategy** | **c** |  |  | **c** |  |
| **Reporting Guideline** | **c** |  |  | **c** |  |
| **Background** |  |  |  |  |  |
| **Related to literature** | **c** | **+** |  | **c** |  |
| **Methodology** | **c** | **+** |  | **c** |  |
| **Results** | **+** | **+** |  |  |  |
| **Conclusions** | **+** | **+** |  |  |  |
| **Implication for research** | **c** |  |  | **c** |  |
| **Implication for practice/society** | **c** | **+** |  | **c** |  |
| **Communication** | **c** | **+** |  | **c** |  |
| **References** |  |  |  |  |  |
| **Comments to editor** | **yes** | **no** |  |  |  |
| **Comments to author** | **yes** | **yes** |  |  |  |
| **Decision** | **Minor** | **Accept** | **Minor** |  | **Accept** |

| **Manuscript 12.** | **MPR**  **R0** | **SPR1**  **R0** | **SPR2**  **R0** | **Editor**  **R0** | **Author**  **R1** | **MPR**  **R1** | **SPR1**  **R1** | **Editor**  **R1** |
| --- | --- | --- | --- | --- | --- | --- | --- | --- |
| **Title** |  |  |  |  |  | **c** |  |  |
| **Article type** |  |  |  |  |  |  |  |  |
| **Abstract** | **c** |  |  |  |  | **c** |  |  |
| **Originality** |  | **c** | **+** |  | **c** | **+** |  |  |
| **Type of review** |  |  |  |  |  | **c** | **c** |  |
| **Search strategy** | **c** | **c** |  |  | **c** | **c** | **c** |  |
| **Reporting Guideline** | **c** |  |  |  | **c** | **c** | **c** |  |
| **Background** | **c** |  |  |  | **c** |  |  |  |
| **Related to literature** |  | **c** | **+** |  | **c** | **+** | **c** |  |
| **Methodology** | **c** | **c** | **+** |  | **c** | **-** | **c** |  |
| **Results** | **c** | **c** | **c** |  | **c** | **-** | **-** |  |
| **Conclusions** | **c** |  |  |  |  |  | **-** |  |
| **Implication for research** |  | **c** | **c** |  | **c** |  |  |  |
| **Implication for practice/society** |  | **c** | **c** |  | **c** | **+** | **-** |  |
| **Communication** |  | **c** | **c** |  | **c** | **c** | **c** |  |
| **References** |  |  |  |  |  |  |  |  |
| **Comments to editor** | **no** | **yes** | **yes** |  |  | **yes** | **yes** |  |
| **Comments to author** | **no** | **yes** | **yes** |  |  | **no** | **yes** |  |
| **Decision** | **Major** | **Major** | **Minor** | **Major** |  | **RR** | **RR** | **RR** |

| **Manuscript 13.** | **MPR1**  **R0** | **MPR2**  **R0** | **SPR1**  **R0** | **Editor**  **R0** | **Author**  **R1** | **MPR1**  **R1** | **SPR1**  **R1** | **Editor**  **R1** |
| --- | --- | --- | --- | --- | --- | --- | --- | --- |
| **Title** |  |  |  |  |  |  |  |  |
| **Article type** |  |  |  |  |  |  |  |  |
| **Abstract** |  |  | **c** |  | **c** |  |  |  |
| **Originality** | **+** | **+** | **+** |  |  |  | **+** |  |
| **Type of review** |  | **+** | **+** |  |  | **+** | **+** |  |
| **Search strategy** |  | **c** | **c** |  | **c** |  |  |  |
| **Reporting Guideline** |  | **c** |  |  | **c** |  |  |  |
| **Background** |  |  | **c** |  | **c** |  | **c** |  |
| **Related to literature** | **c** | **c** | **c** |  | **c** | **+** |  |  |
| **Methodology** | **-** | **+** |  |  |  | **+** |  |  |
| **Results** | **+** | **c** | **c** |  | **c** | **+** | **+** |  |
| **Conclusions** | **+** | **c** |  |  | **c** | **+** |  |  |
| **Implication for research** | **+** | **+** |  |  |  | **+** |  |  |
| **Implication for practice/society** | **+** | **+** | **+** |  |  | **+** | **+** |  |
| **Communication** | **+** | **+** | **+** |  |  | **+** |  |  |
| **References** |  |  |  |  |  |  |  |  |
| **Comments to editor** | **no** | **yes** | **yes** |  |  | **yes** | **yes** |  |
| **Comments to author** | **no** | **yes** | **yes** |  |  | **yes** | **yes** |  |
| **Decision** | **Major** | **Major** | **Major** | **Major** |  | **Accept** | **Minor** | **Accept** |
